# Evaluation of a Microhaplotype-Based Noninvasive Prenatal Test in Twin Gestations: Determination of Paternity, Zygosity, and Fetal Fraction

**DOI:** 10.3390/genes12010026

**Published:** 2020-12-27

**Authors:** Zhaochen Bai, Hu Zhao, Shaobin Lin, Linhuan Huang, Zhiming He, Huan Wang, Xueling Ou

**Affiliations:** 1 Faculty of Forensic Medicine, Zhongshan School of Medicine, Sun Yat-sen University, Guangzhou 510080, China; baizhch@mail2.sysu.edu.cn (Z.B.); zhaohu3@mail.sysu.edu.cn (H.Z.); 2 Guangdong Province Translational Forensic Medicine Engineering Technology Research Center, Sun Yat-sen University, Guangzhou 510080, China; 3 Fetal Medicine Center, Department of Obstetrics and Gynecology, The First Affiliated Hospital of Sun Yat-sen University, Guangzhou 510080, China; shaobin0216@163.com (S.L.); quanquan9811@126.com (L.H.); hezhim5@mail.sysu.edu.cn (Z.H.); 4 Department of Gynecology, Third Affiliated Hospital of Sun Yat-sen University, Guangzhou 510630, China

**Keywords:** microhaplotype, non-invasive prenatal testing, twins, paternity, zygosity, fetal fraction

## Abstract

As a novel type of genetic marker, the microhaplotype has shown promising potential in forensic research. In the present study, we analyzed maternal plasma cell-free DNA (cfDNA) samples from twin pregnancies to validate microhaplotype-based noninvasive prenatal testing (NIPT) for paternity, zygosity, and fetal fraction (FF). Paternity was determined with the combined use of the relMix package, zygosity was evaluated by examining the presence of informative loci with two fetal genome complements, and FF was assessed through fetal allele ratios. Paternity was determined in 19 twin cases, among which 13 cases were considered dizygotic (DZ) twins based on the presence of 3~10 informative loci and the remaining 6 cases were considered monozygotic (MZ) twins because no informative locus was observed. With the fetal genomic genotypes as a reference, the accuracy of paternity and zygosity determination were confirmed by standard short tandem repeat (STR) analysis. Moreover, the lower FF, higher FF, and combined FF in each DZ plasma sample were closely related to the estimated value. This present preliminary study proposes that microhaplotype-based NIPT is applicable for paternity, zygosity, and FF determination in twin pregnancies, which are expected to be advantageous for both forensic and clinical settings.

## 1. Introduction

In recent years, the identification of fetal cell-free DNA (cfDNA) in the maternal circulation has facilitated noninvasive prenatal testing (NIPT) for investigating monogenic diseases [1], identifying aneuploidies [2], and determining paternity [3,4,5,6]. However, conventional prenatal screening options are less robust for twin pregnancies than for singletons [7], yet the miscarriage risk associated with invasive diagnosis is higher among twin pregnancies than singleton pregnancies [8]. According to Hellin’s law, established in 1895, the incidence of twin pregnancies should be one per 30 live births on average [9], but this value has increased with the progressive rise in the average maternal age at the time of conception and the increasingly frequent use of ovulation drugs [10,11,12]. Twin pregnancies are classified as monozygotic (MZ, resulting from division of an embryo into two identical embryonic structures) and dizygotic (DZ, resulting from fertilization of two ova by two sperm). In general, cfDNA testing in twin pregnancies is more complex than in singleton pregnancies because the two fetuses could be either MZ, and therefore genetically identical, or DZ, where they are likely to have different genotypes.

In the field of forensic science, the validation of single-nucleotide polymorphism (SNP)-based NIPT in twin pregnancies and the performance to assign zygosity and paternity have been reported in several studies previously [13,14,15]. However, due to the biallelic nature of SNP markers that limits their per-locus power, a large number of loci are required to ensure reasonable discriminatory power, which results in high costs and the challenge of physical linkage among individual loci. Moreover, the bioinformatic model utilized for paternity determination was based on the hypothesis that the twin fetuses had the same biological father, ignoring the probability of heteropaternity superfecundation (HS) among DZ twins, which has a reported frequency of 2.4% in paternity suits [16]. Hence, a more discriminative genetic marker with a reliable strategy for data interpretation is needed for prenatal paternity analysis in twin pregnancies.

The microhaplotype, a novel type of genetic marker, refers to a locus with two or more closely linked SNP loci in a short segment of DNA (generally < 200 bp) that generates more than three alleles and exhibits discriminating power comparable to short tandem repeats (STRs) [17]. Additionally, the absence of stuttered artifacts and imbalanced heterozygous alleles has facilitated microhaplotype markers that are advantageous in the forensic DNA mixture interpretation [18,19,20,21]. However, little is known about the additional benefits of microhaplotypes in NIPT. In our previous study, a solution-based, high-redundancy DNA probe capture assay targeting 60 microhaplotypes was developed [22], demonstrating desirable performance in cfDNA sequencing and subsequent relationship interference in singleton pregnancies [23]. In this preliminary study, we evaluate the performance of microhaplotype-based NIPT to assign paternity, zygosity, and fetal fraction in twin gestations.

## 2. Materials and Methods

### 2.1. Sample Collection and Preparation

Five milliliters of peripheral blood were derived from 19 pregnant women who had signed consent forms. All samples were from twin pregnancies confirmed by ultrasound scanning. Peripheral blood samples from pregnant women from 10 weeks of gestation onwards were collected into K3-EDTA tubes. Moreover, dual amniocentesis was performed among twin pairs from each amniotic cavity at 16 to 24 weeks of gestation, and 2 ML blood samples from the husbands (alleged fathers of the twins) were collected. Maternal blood samples were then subjected to two sequential rounds of centrifugation at 1600× *g* and then at 16,000× *g* within 8 h to isolate maternal plasma first and then remove the remaining white blood cells (WBCs). cfDNA from maternal plasma samples and genomic DNA (gDNA) from the blood cells were extracted as previously described [24]. Moreover, the gDNA samples from the paternal blood samples and the fetal tissues were also extracted for reference [24]. The obtained cfDNA and gDNA were stored at −20 °C until further assessment. All subjects, including the pregnant women and the alleged fathers, were fully aware of the purpose, nature, method and possible adverse reactions. They were informed the risks of taking part in the present study and the associated anxiety that might arise from the possible conclusion of non-paternity. Afterwards, all subjects gave their informed consent for inclusion before they participated in the study. The study was conducted in accordance with the Declaration of Helsinki, and the experimental protocol was approved by the Ethics Committee of Zhongshan School of Medicine, Sun Yat-sen University (protocol code 2019-062, date of approval 10 June 2019), on behalf of the Ethical Review Board of Sun Yat-sen University.

### 2.2. Library Preparation, Sequencing, and Data Analysis

Both extracted cfDNA and gDNA were used to prepare the DNA library using a custom designed MPS-based assay targeting 60 microhaplotypes as previously described [22]. The purified DNA library was sequenced on a HiSeq 2000 sequencer (Illumina) with a read length of 150 bp for each end, according to the standard paired-end protocol. After sequencing, paired end read fragments of each sample were processed using in-house quality control software to remove adaptor sequences and poor-quality reads. The resulting FASTQ sequences were aligned to the reference genome hg19 (human_g1k_v37.fasta) using the Burrows–Wheeler alignment algorithm [25]. Sambamba tools were used to remove duplicate reads [26]. Microhaplotype alleles were assigned by an in-house Excel-based workbook as previously described [23].

### 2.3. Calculation of Likelihood Ratios (LRs)

Regarding relationship interference in twin pregnancies, the weight of evidence was assessed by likelihood ratios (LRs) that consider a number of mutually competing hypotheses as follows:

**Hypothesis 1** **(H1).** The alleged father (AF) is the biological father of both twin fetuses.

**Hypothesis 2** **(H2).** AF and a random man are the biological fathers of the twin fetuses (e.g., HS).

**Hypothesis 3** **(H3).** A random man or two random men are the biological fathers of the twin fetuses.

We could then define likelihood ratios (LRs) using the hypotheses listed above. We let *LR*_H1,H3_, *LR*_H2,H3_, and *LR*_H1,H2_ be the LRs for hypotheses H_1_ vs. H_3_, H_2_ vs. H_3_, and H_1_ vs. H_2_, respectively. The LR values were then calculated using the R package relMix (http://cran.r-project.org/web/packages/relMix) [27,28]. A drop-in probability *c* of 0.01 and mutation rate *r* = 10^−8^ were chosen, with silent allele *s* and theta correction *θ* set to 0. Allele frequency data for LR calculation are shown in our previous study [23], and a frequency of 0.002 ($\frac{1}{208\times 2+1}$) was applied for alleles not included in the data set.

### 2.4. Assessment of Zygosity and Fetal Fraction

**Figure 1:**
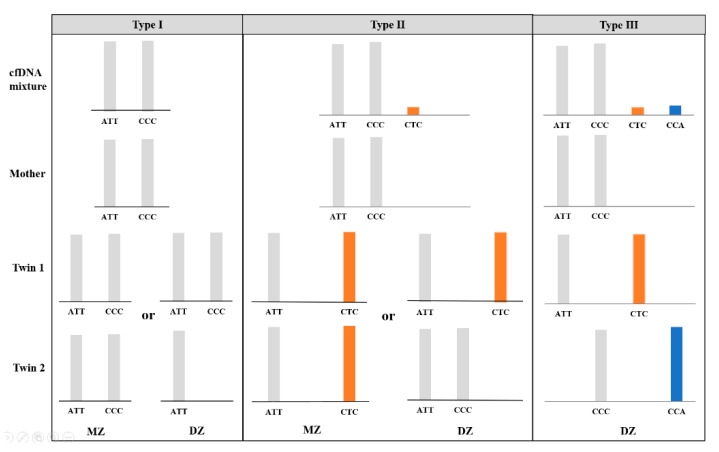
Three possible types of microhaplotype patterns (type Ι, ΙI and III) in the cfDNA mixture attained from twin pregnancies and examples of genotypic combinations of parents and offspring, without consideration of allele dropout and drop-in. The presence of a “Type III” pattern is considered informative for a dizygotic (DZ) pregnancy.

In theory, every child inherits one allele from its mother (maternal allele) and one from its father (paternal allele). With the maternal genomic sequencing results as a reference, nonmaternal alleles in the plasma sample could be considered paternal alleles when the allele fraction satisfied a threshold of 1.0%. For an MZ pregnancy where the twin fetuses are genetically identical, the interpretation of the microhaplotype pattern is essentially the same as for a singleton pregnancy; thus, no more than one paternal allele was expected in the maternal plasma at a single locus (“Type 1” and “Type 2”). For a DZ pregnancy, the nonmaternal alleles are interpreted as being derived from both fetuses. In addition to the possible presence of “Type 1” and “Type 2” loci, two additional nonmaternal alleles at one locus were assumed to be present (“Type 3”) when the twins inherited two distinct paternal alleles that were different from the maternal alleles (Figure 1). Therefore, we consider the “Type 3” microhaplotype as an informative locus indicating a DZ pregnancy.

Furthermore, the fetal fraction (FF) in maternal cfDNA was determined based on the allele ratios. For MZ twins, a combined FF was estimated with the same methodology as for singletons, and for DZ twins, two distinct assumed paternal alleles were utilized for individual FF calculation. As previous studies have shown that each fetus in a DZ gestation contributes different amounts of cfDNA in maternal circulation, the difference can be nearly 2-fold. Assuming that the fetus-specific allele with the lower sequencing depth at each informative locus was constantly derived from the fetus with lower FF and vice versa, the fetal allele with higher depth was derived from the fetus with the higher FF, and the averaged lower FF value *FF* (*l*) or higher FF value *FF* (H) was therefore estimated with the following equations:

(1) $$\bar{FF\left(l\right)}=\frac{1}{N}\overset{N}{\underset{i=1}{\sum }}FF{\left(l\right)}_{i}=\frac{1}{N}\overset{N}{\underset{i=1}{\sum }}\frac{2\times d{\left(l\right)}_{i}}{D_{i}}$$

and,

(2) $$\bar{FF\left(h\right)}=\frac{1}{N}\overset{N}{\underset{i=1}{\sum }}FF{\left(h\right)}_{i}=\frac{1}{N}\overset{N}{\underset{i=1}{\sum }}\frac{2\times d{\left(h\right)}_{i}}{D_{i}}$$

where *d* (*l*)*i* or *d* (H)*i* represents the fetus-specific allele with the lower or higher sequencing depth at the informative locus *i*, *D_i_* denotes the total sequencing depth of locus *i*, and *N* is the total number of informative loci observed in a DZ maternal cfDNA sample.

### 2.5. Conventional Genotyping of Short Tandem Repeat (STR) Markers

As a separate experiment, conventional STR typing was performed to confirm the paternity and zygosity of each family case using invasive samples from each twin fetus with the Goldeneye^®^ DNA ID 25A system according to the manufacturer’s protocols. Based on the autosomal STR genotyping results, PI and CPI were calculated following the Chinese national standards recommended for paternity testing (GB/T 37223-2018) to determine paternity.

## 3. Results

### 3.1. Paternity Determination

**Figure 2:**
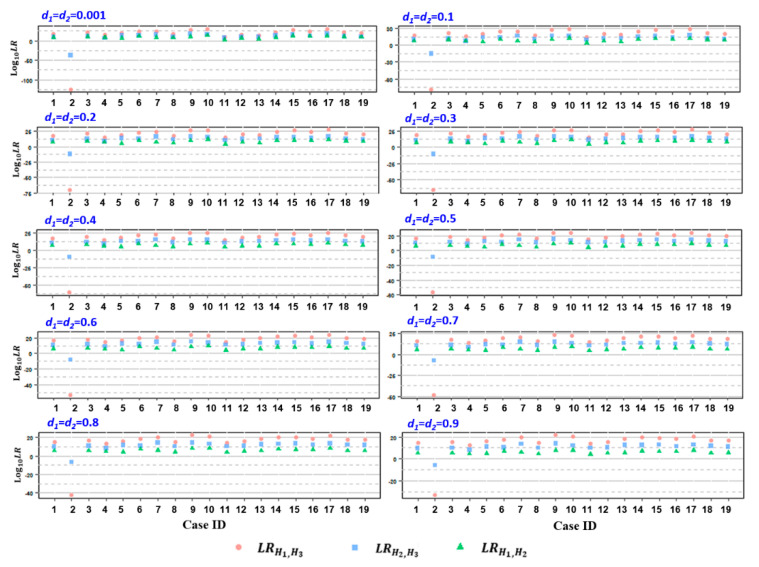
The distribution of log_10_LRs, including *LR*_H1,H3_ (pink circle), *LR*_H2,H3_ (blue square), and *LR*_H1,H2_ (green triangle), attained from the 19 studied cases, assuming equal dropout probability *d* for both twins. *d*_1_ and *d*_2_ represent the individual *d* values of the twin fetus, ranging from 0.001~0.9.

To determine paternity, the relMix package was used to calculate the LRs that considered a few competing hypotheses as described above for each microhaplotype locus for each alleged father. Cumulative LR was attained by multiplying the individual LR values based on previous observations that the 60 loci are independent of each other [23]. As a result, the attained *LR*_H1,H3_ and *LR*_H1,H2_ values were all above 100 (in log_10_, ranging from 2.212 to 30.001) for 18 cases except case 2, with an assumed equal dropout probability *d* for both twins (ranging from 0.001 to 0.9), while the calculated values of *LR*_H1,H3_ and *LR*_H2,H3_ for case 2 were all below 1 (Table S1, Figure 2), indicating the inclusion of *AFs* as a biological father in relation to both twins in 18 cases but the exclusion of *AF* with regard to both twins in case 2. Similar results were attained when the dropout probability was assumed to be different between the twins (Table S2, Figure S1). Subsequent standard STR analysis by means of amniocentesis at 18 weeks of gestation confirmed *AFs* as the biologic father of both twins in 18 cases (combined paternity index (CPI) ranging from 7.451 to 17.682 in the log_10_ form) (Table S3). In contrast, for *AF* in case 2, the paternity of both twins was excluded based on 10 and 17 discordant loci out of 23 autosomal STRs tested, respectively (Table S3).

The specificity of the paternity test was examined by testing 98 unrelated males as alleged fathers in two example cases (case ID: 2 and 16), whose genotypes were retrieved from the 1000 Genomes Project [29]. The resulting *LR*_H1, H3_ and *LR*_H2, H3_ values of the 98 unrelated males were below 1 as an equal dropout probability in a reasonable range (Tables S4 and S5, Figure S2). However, when the assumed *d* values were unrealistically high (e.g., *d* ≥ 0.5), a fraction of unrelated subjects (4 in case 2 and 1 in case 16) exhibited *LR*_H2,H3_ values above 1, which may result in a misleading conclusion (Table S4, Table S5, Figure S2).

### 3.2. Zygozity Evaluation and Fetal Fraction Quantification

With the maternal DNA profile obtained from peripheral blood cells in each case as the reference, there were 31~46 nonmaternal alleles (fetus-specific) with allele ratios ≥ 1.0% at the 60 targeted microhaplotypes in the 19 studied cases, among which 3~10 informative loci with two additional nonmaternal alleles, indicating DZ twin pregnancy, were detected in 13 maternal cfDNA samples (case ID: 2, 3, 5, 6, 7, 8, 9, 10, 12, 14, 15, 16, and 17) (Table 1). In contrast, the remaining 6 plasma samples (case ID: 1, 4, 11, 13, 18, and 19) always contained at most one additional nonmaternal microhaplotype allele at a single locus. These data suggested that 6 cases were MZ twin pregnancies (Table 1). Example of microhaplotype genotyping profiles of DZ twin pregnancy (case ID: 2) is shown in Table S6. As a separate experiment, we compared the genotypes across the 23 STRs for the invasive fetal samples from each twin pair to ascertain whether the twins were MZ or DZ and attained consistent results with those from the noninvasive microhaplotype-based approach (Table S3).

For MZ twins, a combined FF was estimated with the same methodology as for singletons and ranged from 9.34% to 18.08% (Table 1). For DZ twins, two individual FFs were quantitated using two fetal-specific alleles at each informative locus, assuming that the fetal allele with the lower or higher sequencing depth was constantly derived from the fetus with the lower or higher FF value. As a result, the calculated lower FF ranged from 2.99% to 8.01%, and the higher FF ranged from 5.40% to 20.29%, with the combined FF ranging from 8.43% to 27.58% in the 13 assumed DZ cases (Table 1).

### 3.3. Validation with Microhaplotype Sequencing of Fetal Tissues

The microhaplotype sequencing results of the fetal gDNA samples were retrieved and served as references for the identification of real fetus-specific alleles (paternally inherited) in the maternal cfDNA. The results demonstrated that 94.44%–100.00% of actual fetal alleles were successfully identified based on the predefined threshold (1.0%), with 0–2 dropout alleles and 0–2 drop-in alleles. Moreover, the actual number of DZ informative loci with two distinct fetal alleles was 3–10 in the 13 DZ cases, whereas none were present in the remaining 6 MZ cases (Table 1). Additionally, for each marker, we counted the number and percent of informative loci for DZ twin pregnancy. The results showed that 45 loci (45/60 = 75%) were informative loci in at least 1 DZ case and that the percentage informativity for each microhaplotype ranged from 0% to 61.54% (Table S7). It should be noted that locus MH-32, which has the highest effective number of alleles (Ae) based on our previous study, also has the highest percentage of informativity for DZ pregnancy (61.54%) (Table S7).

**Figure 3:**
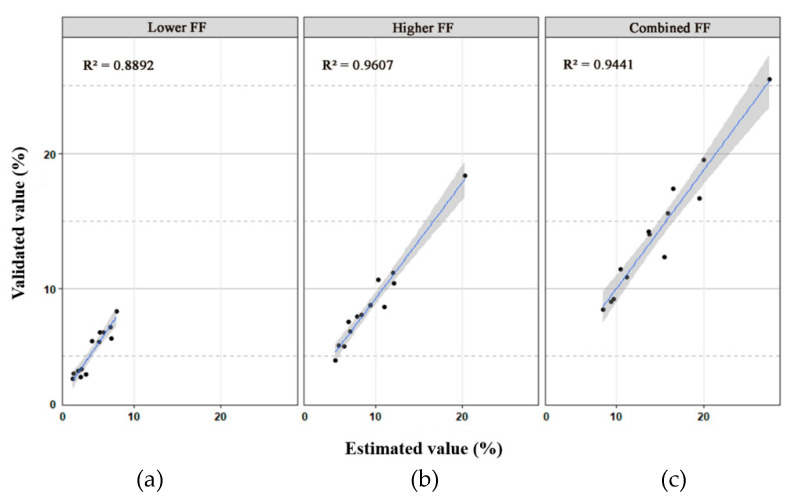
Scatter plots with 95% confidence intervals of the validated value and estimated value of the lower fetal fraction (FF) (**a**), higher FF (**b**), and combined FF (**c**) for 13 dizygotic (DZ) twin cases. The coefficient of determination (R-square) is also shown in the upper left corner of each plot.

With the fetal genomic genotypes as a reference, the lower FF, higher FF, and combined FF in each DZ plasma sample were revealed to be 3.33%–8.33%, 4.71%–18.33%, and 8.44%–25.50%, respectively (Table 1), which were closely related to the estimated value (R^2^ = 0.889, 0.960 and 0.944, respectively) (Figure 3). Accordingly, the difference in cfDNA amounts contributed by each fetus in each DZ twin pair varied from 1.264- to 2.557-fold (Table 1). However, the combined FF in MZ cases varied from 9.04% to 18.08%.

## 4. Discussion

As a preliminary study, we report the validation of microhaplotype-based NIPT methodology to determine paternity, assign zygosity, and evaluate fetal fraction in maternal plasma as early as 10 weeks of gestation in twin pregnancies. With the combination of the R package relMix for data interpretation, the paternity of 19 twin fetuses was successfully determined in a noninvasive way without prior knowledge of zygosity. In contrast to conventional PI calculations that consider paternity or nonpaternity, the kinship LR calculation in the present study was based on multiple competing hypotheses, including the probabilities of the alleged father being the biological father of both, one, or none of the twin fetuses. Moreover, a total of 98 unrelated men were excluded as alleged fathers in 2 sample cases when the dropout probabilities were in a reasonable range of values, which further verified the specificity of the approach. It should be noted that, as a qualitative model, the R package relMix has been validated in kinship analysis in NIPT [22,27,28] and other mixed DNA [30], evaluating the questioned relationship by incorporating a number of stochastic effects (allele dropout, drop-ins, mutation, etc.). However, when the assumed *d* values were unrealistically high, this model considered the mismatches between the unrelated males and the cfDNA mixture to be caused by dropout rather than nonpaternity, which may result in a false inclusion of paternity. Further study is needed to incorporate more polymorphic markers to ensure sufficient discriminative power and to establish a dropout probability prediction model for twin pregnancies. Additionally, based on the previously reported combined FF value in twin pregnancies (ranging from approx. 7% to 35%) [31], we did not suppose dropout probability *d* = 0 for individual fetus because it is fallacious for the minor contributors of cfDNA mixtures. Instead, we set the min. *d* value to 0.001 for individual fetus in line with the error rate of current MPS platform (approx. 0.1%) [32]. In contract, we let *d* = 0 for the maternal portion (major contributors of cfDNA).

We also report on the performance of microhaplotype-based NIPT in zygosity determination, where a paternal sample was not required. Knowledge of the zygosity status for evaluation of paternity in twin pregnancy is advantageous because the risk of heteropaternity superfecundation (HS) could be potentially eliminated when the zygosity status is diagnosed as monozygotic, indicating that the twins are genetically identical. Likewise, accurate determination of zygosity is also clinically important, particularly when chorionicity remains unconvincing based on an early ultrasound examination. Indeed, monochorionic (MC) twins share their blood supply, and up to 15% are affected by twin-to-twin transfusion syndrome (TTTS) [33] or other related abnormalities; therefore, establishing dizygosity could help exclude the occurrence of these abnormalities, because DZ twins almost always have a dichorionic (DC) placenta, and then provide aid in patient management. Several studies have shown the feasibility of noninvasive zygosity determination by using biallelic SNP markers [13,15,34]; however, this method requires relatively large amounts of SNPs, reaching up to thousands [15] or even ten thousands [13,34] of loci, which will result in high costs and complicated data interpretation. Recently, Dziennik et al. employed STRs and deletion/insertion polymorphism (DIP)-STR compounds as tools for zygosity determination by detecting fetal alleles in maternal cfDNA [35]. A certain limitation of this assay is the risk of a false positive result (false detection of DZ pregnancy) caused by the stutters produced during the amplification of STRs or DIP-STRs. In addition, the DIP-STR-based methods were also restricted by various combinations of maternal-twin genotypes. In contrast, without the burden of stutter artifacts, the novel multiallelic microhaplotypes may enable straightforward detection of fetal alleles in cfDNA that yield a more accurate determination of zygosity in the present study. As suggested by Kidd et al., expectations for the ability of mixture deconvolution are given for different integer values of the effective number of alleles (Ae) at a locus [36,37]. Herein, we observed that the locus with the highest Ae value [23] also possesses the highest percentage informativity for a DZ pregnancy.

Additionally, the estimation of fetal fraction (FF), including combined FF for MZ twins and individual FFs for DZ twins, is an equally important determinant of the performance of NIPT for both forensic and clinical settings. For a DZ pregnancy, studies have shown that the circulating fetal DNA from both twins can vary by nearly 2-fold [38] (up to 2.558-fold in the present dataset); accordingly, it is possible that the lower FF may be too limited for reliable analysis. In this situation, if NIPT measures only a combined FF in a DZ twin pregnancy, especially when only one fetus with the lower FF contribution was involved in nonpaternity or aneuploidy, it is likely to come to an erroneous conclusion [34]. Herein, the present study describes a microhaplotype-based FF quantification assay by directly analyzing fetus-specific microhaplotype alleles at informative loci in DZ cfDNA samples. This conservative estimation ensures that the possible lowest FF of each sample is considered as a quality control of the performance of NIPT. A certain limitation of our present assays is that the number of informative loci was quite limited in some studied cases (only 3 loci in cases 8 and 12), which further illustrates the need for more polymorphic microhaplotype markers with higher Ae values.

## 5. Conclusions

In summary, the targeted test described here constitutes an integrated microhaplotype-based assay that incorporates simultaneous determination of paternity, zygosity, and fetal fraction based on the same specimen (maternal cfDNA mixture) in twin pregnancies, as a preliminary study. The algorithm for paternity analysis uses a Bayesian approach, and additional information such as stochastic effects or mutation rate can be easily incorporated into the model. Moreover, the microhaplotype-based approach is also expected to be advantageous in zygosity assignment and individual FF estimation for DZ pregnancies because the absence of stutter burden has made microhaplotypes a more straightforward tool for the detection of fetal alleles and therefore improves the estimation of nonpaternity or aneuploidy risk because the prior risk is different based on zygosity status. Additionally, the detection threshold of individual lower FF for DZ twins (or combined FF for MZ) should also be considered as a quality control of the performance of NIPT before making a confident conclusion. Further investigations based on larger samples and more sophisticated validation are required to determine the minimum number of microhaplotype markers in the total dataset as well as the minimum number of informative loci for data interpretation in DZ pregnancies.

## Figures and Tables

**Table 1:** Detailed information for the analysis of fetus-specific microhaplotype alleles (paternal alleles) in cfDNA attained from 19 twin pregnancies.

Case ID	cfDNA								Validated by Fetus Tissue							
	*n.* of Locus *	*n.* of Locus **	*n.* of Assumed PA	*Est.* Lower FF %	*Est.* Higher FF %	*Est.* Combined FF %	*n.* of Locus †	*n.* of Locus ‡	*n.* of Real PA ††	*n.* of PA Dropout	*n.* of Drop-In	PA Detection Rate	Lower FF %	Higher FF %	Combined FF %	Ratio §
2	37	8	45	5.21	8.53	13.74	37	8	45	0	0	100%	6.13	8.06	14.18	1.316
3	33	3	36	3.84	5.81	9.65	33	3	36	0	0	100%	3.43	5.82	9.25	1.695
5	36	6	42	5.94	7.88	13.82	36	5	41	0	1	100%	6.08	7.95	14.03	1.308
6	37	8	45	6.13	10.35	16.48	37	8	45	0	0	100%	6.77	10.62	17.40	1.569
7	37	6	43	7.28	20.29	27.58	37	6	43	0	0	100%	7.17	18.33	25.50	2.558
8	31	3	34	4.51	11.02	15.53	32	4	36	2	0	94.44%	3.66	8.66	12.32	2.369
9	40	6	46	3.53	6.88	10.41	41	6	47	1	0	97.87%	3.90	7.52	11.42	1.928
10	36	10	46	8.01	12.02	20.03	36	10	46	0	0	100%	8.33	11.18	19.51	1.341
12	37	3	40	7.35	12.14	19.49	36	3	39	0	1	100%	6.30	10.37	16.67	1.646
14	36	7	43	6.46	9.47	15.93	36	7	43	0	0	100%	6.75	8.79	15.54	1.301
15	37	6	43	3.03	5.40	8.43	37	5	42	0	1	100%	3.72	4.71	8.44	1.265
16	34	6	40	4.00	7.16	11.16	34	6	40	0	0	100%	4.04	6.81	10.85	1.685
17	39	7	46	2.99	6.40	9.39	40	7	47	1	0	97.87%	3.33	5.70	9.03	1.715
1	33	0	33	NA	NA	11.90	33	0	33	0	0	100%	NA	NA	11.90	NA
4	31	0	31	NA	NA	15.96	31	0	31	0	0	100%	NA	NA	15.96	NA
11	33	0	33	NA	NA	9.05	33	0	33	0	0	100%	NA	NA	9.05	NA
13	38	0	38	NA	NA	18.08	38	0	38	0	0	100%	NA	NA	18.08	NA
18	31	0	31	NA	NA	9.34	31	0	31	0	0	100%	NA	NA	9.34	NA
19	34	0	34	NA	NA	11.90	34	0	34	0	0	100%	NA	NA	11.90	NA

*: locus with at least one assumed paternal allele (PA) observed in maternal cfDNA. **: locus with two assumed PAs observed in cfDNA, e.g., informative locus for a DZ pregnancy. †: locus with at least one real PA that should be detected in cfDNA without consideration of dropout and drop-in. ‡: locus with two real PAs that should be detected in cfDNA without consideration of dropout and drop-in. ††: the maximum number of PAs that should be detected in cfDNA without consideration of dropout and drop-in. §: higer fetal fraction (FF) vs. lower FF in DZ cfDNA. NA: not applicable.

## Data Availability

The data presented in this study are available on request from the corresponding author. The data are not publicly available due to restriction of privacy.

## Supplementary Materials

The following are available online at https://www.mdpi.com/2073-4425/12/1/26/s1, Figure S1: The distribution of log_10_LRs, including *LR*_H1,H3_ (pink circle), *LR*_H2,H3_ (blue square), and *LR*_H1,H2_ (green triangle), attained from 2 examples of cases, with 15 various choices of *d* values that were assumed to be different for the twins, as indicated on the left side. *d*_1_ and *d*_2_ represent the lower and higher *d* values of the twin fetus, respectively, Figure S2: Violin plots illustrating the distribution of *LR*_H1,H3_ (pink dots) and *LR*_H2,H3_ (blue dots) of 98 unrelated males attained from 2 examples of cases, with an assumed equal dropout probability *d* (0.001~0.9) for both twins. Rugplots display the distributions of *LR*_H1,H3_ (pink, left) and *LR*_H2,H3_ (blue, right) values in the whole dataset, Table S1: The value of Log_10_(*LR*_H1,H3_), Log10(*LR*_H2,H3_), and Log10(*LR*_H1,H2_) attained from the 19 studied cases, assuming equal dropout probability *d* for both twins. *d*1 and *d*2 represent the individual *d* values of the twin fetus, ranging from 0.001~0.9, Table S2: The value of Log_10_(*LR*_H1,H3_), Log10(*LR*_H2,H3_), and Log10(*LR*_H1,H2_) attained from 2 examples of cases (case ID: 2 and 16), with 15 various choices of *d* values, Table S3: The paternity analysis of the alleged fathers and zygosity determination in each twin pregnancies using STR-based conventional assay with invasive fetal samples, Table S4: The value of Log_10_(*LR*_H1,H3_) and Log10(*LR*_H2,H3_) of 98 unrelated males attained from case 2, with an assumed equal dropout probability *d* (0.001~0.9) for both twins, Table S5: The value of Log_10_(*LR*_H1,H3_) and Log10(*LR*_H2,H3_) of 98 unrelated males attained from case 16, with an assumed equal dropout probability *d* (0.001~0.9) for both twins, Table S6: Example distribution of nonmaternal microhaplotype alleles observed in a DZ twin case (case ID: 2), Table S7: The calculation of percentage informativity for dizygotic (DZ) pregnancies and effective number of alleles (Ae) for each microhaplotype.
