# Supplementary figures and images for: Evaluation of a Microhaplotype-Based Noninvasive Prenatal Test in Twin Gestations: Determination of Paternity, Zygosity, and Fetal Fraction
Source: Genes (Basel). 2020 Dec 27;12(1):26. doi: 10.3390/genes12010026 (PMC7823673; doi:10.3390/genes12010026)

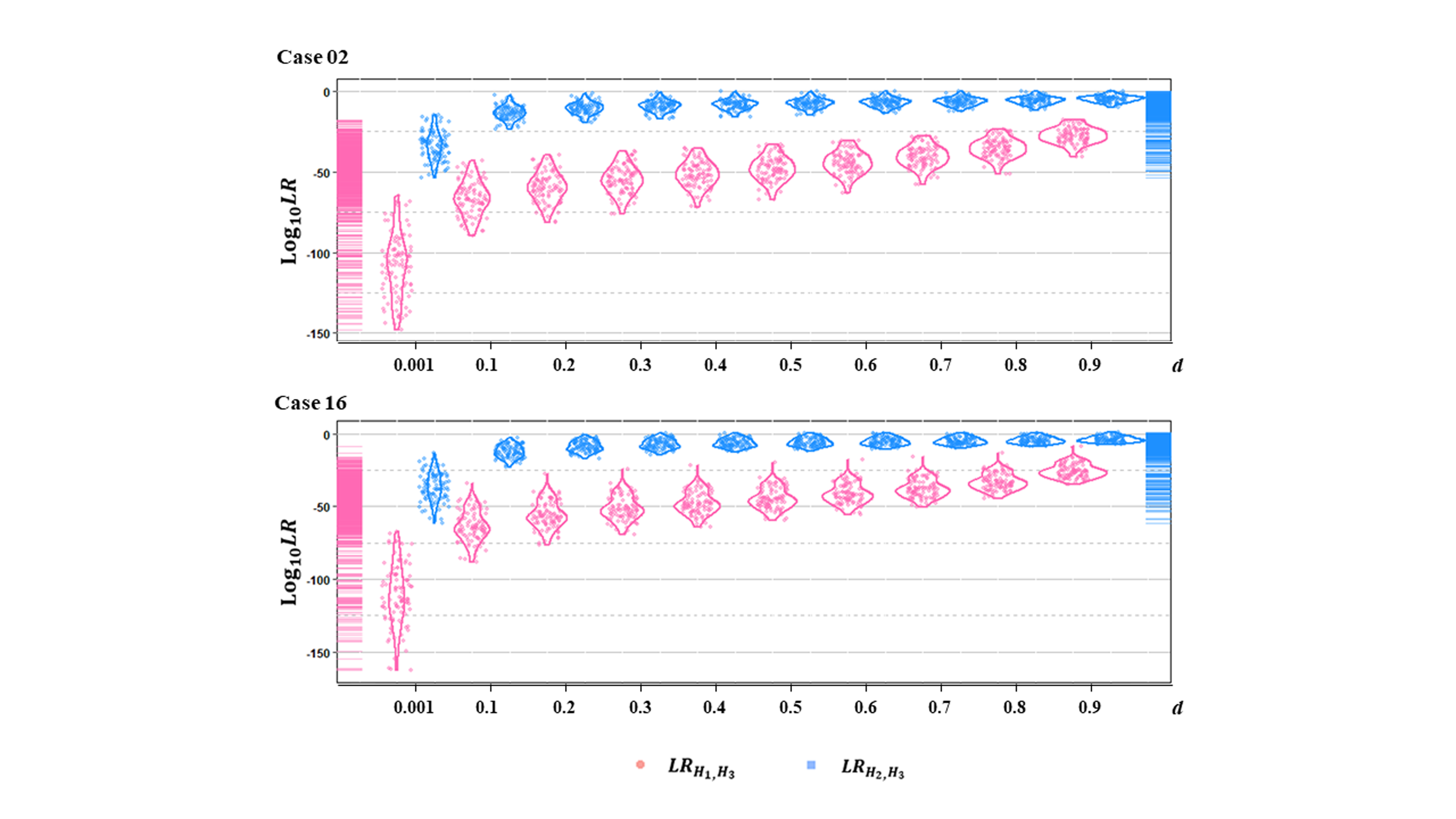

Supplement: Supplementary file 1 [file genes-12-00026-s001.zip › Supplementary Files/Figure S2-300.tif]

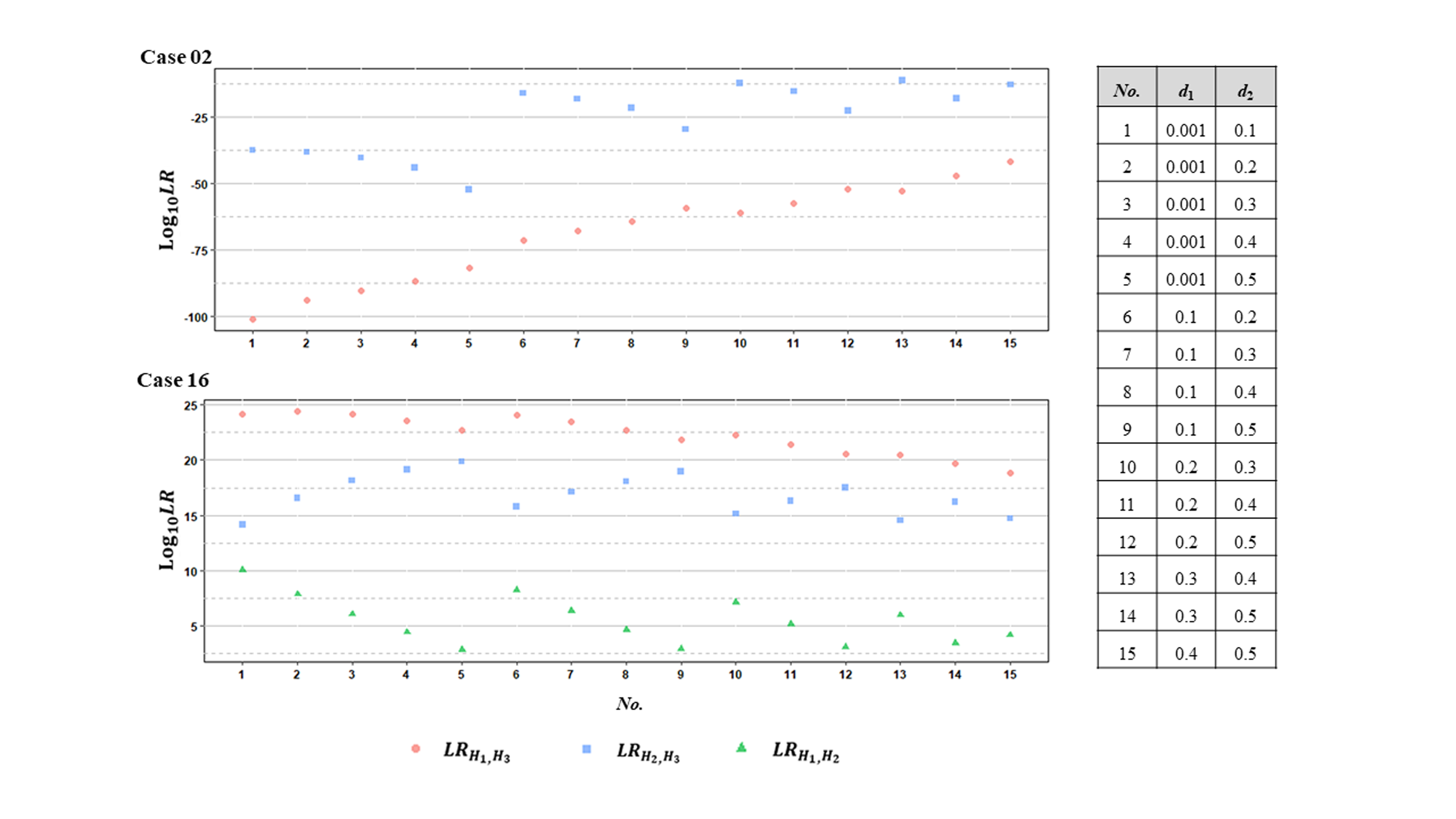

Supplement: Supplementary file 1 [file genes-12-00026-s001.zip › Supplementary Files/Figure S1-300.tif]
